# Impact of Tussigenic Stimuli on Perceived Upper Airway Sensation and Motor Cough Response Following Total Laryngectomy

**DOI:** 10.3389/fphys.2020.00477

**Published:** 2020-05-29

**Authors:** Amy Fullerton, Yuhan Mou, Natalie Silver, Neil Chheda, Donald C. Bolser, Karen Wheeler-Hegland

**Affiliations:** ^1^Department of Communication Sciences and Disorders, Brooks Rehabilitation College of Healthcare Science, Jacksonville University, Jacksonville, FL, United States; ^2^Laboratory of Upper Airway Dysfunction, Department of Speech, Language and Hearing Sciences, University of Florida, Gainesville, FL, United States; ^3^Division of Head and Neck Surgery, Laboratory of Head and Neck Cancer Translational Science, Department of Otolaryngology, University of Florida, Gainesville, FL, United States; ^4^Laboratory of Physiology and Pharmacology of Cough and Airway Protection, Department of Physiological Sciences, University of Florida, Gainesville, FL, United States

**Keywords:** respiratory defensive reflex, capsaicin, laryngectomy, urge to cough, airway protection

## Abstract

**Background:**

Total laryngectomy (TL) is standard intervention for carcinoma of the head and neck or, in cases of non-functional larynx, as a result of disease or radiation exposure. Laryngeal extirpation serves as a unique human model of both recurrent and superior laryngeal nerve section and offers insight into motor and sensory aspects of cough: both volitional and in response to tussigenic stimuli. While motor changes in cough function are expected among those status post-TL due to postoperative reconstruction of the upper airway, motor cough parameters have not been well described and sensory aspects of cough are unknown in this population, which provides insight into a vagal denervation model in humans.

**Methods:**

Data were collected from three groups totaling 80 adults (39 male), including 25 healthy younger adults (HYA), 27 healthy older adults (HOA), and 28 adults post-TL. Cough was elicited both upon command and in response to nebulized capsaicin. Outcome measures included urge to cough and cough airflows.

**Results:**

Kruskal–Wallis test showed that two of the three groups differed significantly by urge to cough χ^2^(2, *N* = 244) = 8.974, *p* = 0.011. *Post hoc* analysis showed that post-TL subjects had reduced perceived urge to cough at all concentrations of capsaicin (*p* < 0.05). Cough airflows were significantly reduced for post-TL subjects compared to healthy controls in all metrics except post-peak phase integral (PPPI) for which HOA and TLs were comparable under both volitional and capsaicin-induced conditions.

**Conclusions:**

These findings support the hypothesis that both cough airflow and sensations are significantly reduced in post-TL subjects when compared with HOA. Interestingly, HOA and post-TL subjects have comparably reduced UTC and cough airflows when compared to HYA. The only metric of cough airflow for which these groups differ is the PPPI, which may be a compensatory adaptation for reduced cough airflows and/or sensation.

## Introduction

Laryngeal neuropathy has been implicated in several conditions with deleterious effects on speech, swallow, and cough. Deficits involving laryngeal somatosensation occur concomitantly with dysphagia (Hammer et al., 2013; Troche et al., 2014) and dysarthria (Hammer and Barlow, 2010) in those with Parkinson’s disease, as well as in chronic cough (Bock et al., 2014), and sensation in the larynx, provided by the superior laryngeal nerve (SLN; Bradley, 2000), is known to decline with age (Aviv, 1997). Stockwell et al. (1993) found that cough elicited by mechanostimulation of laryngeal mucosa was abolished following lidocaine ablation of the SLN but that cough thresholds in response to chemostimulation (e.g., citric acid) were comparable to those with intact SLN input.

**FIGURE 1:**
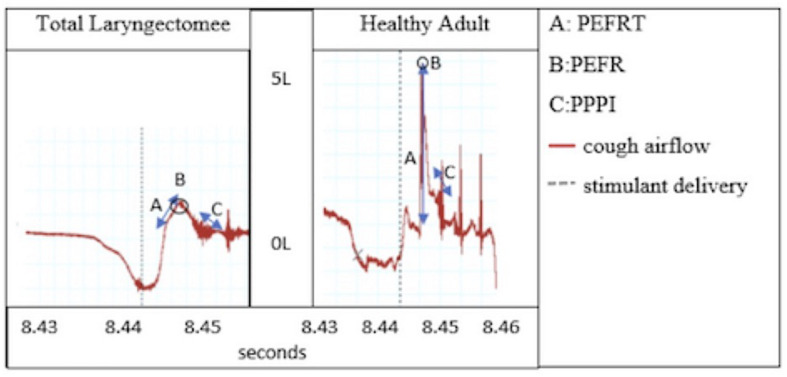
Cough airflow: Peak expiratory flow rise time (PEFRT), Peak expiratory flow rate (PEFR), and post-peak phase integral (PPPI).

This was elaborated upon by Fontana et al. (1999, 2002) who found reduced cough volume acceleration (CVA) due to reduced peak expiratory flow rise times (PEFRT; Figure 1) and comparable cough thresholds in response to nebulized fog inhalation in post-total laryngectomy (TL) subjects and those with Parkinson’s disease, which have been shown to be reduced as compared to healthy controls (Leow et al., 2012; Troche et al., 2014). These findings provide preliminary insight into cough aspects specific to laryngectomy but fail to address the role of sensory input following SLN denervation. Cough thresholds reported thus far have been based on cough response to lowest fog output capable of evoking at least one cough during two distinct challenges separated by a 30-min time interval and are dissimilar from urge to cough, which can be elicited at thresholds lower than that required for motoric cough response.

Total laryngectomy involves complete separation of the upper and lower airways. This surgery entails directly connecting the oral cavity via a tubed neopharynx to the esophagus and creation of a stomal aperture in the neck to enable respiration. Given the surgical separation of upper from lower airways and loss of larynx, this population offers unique insight into the distinct contribution of these structures to respiratory behaviors integral to cough. This population frequently experiences anosmia and dysguesia (Caldas et al., 2013), deficits largely attributed to the separation of airway and alimentary tracts, although viscerosensory deficits unrelated to this surgical revision, such as reduced sensation of thirst (Miyaoka et al., 1987), have also been reported.

The objective of the current study was to determine the sensorimotor characteristics of cough following TL compared to healthy controls. Three populations were used to evaluate urge to cough in response to differing concentrations of a tussigenic stimulus; these included healthy younger adults (HYA), healthy older adults (HOA), and those status post-TL. Given the structural changes post-TL, namely, loss of glottal compression preceding expulsion, cough airflows were also hypothesized to be reduced compared to healthy controls. Further, cough sensitivity, as measured by urge to cough in response to inhalation of a tussigenic stimulus, was hypothesized to be reduced after TL compared to both groups of healthy controls due to lack of laryngeal sensory information in the TL group.

## Materials and Methods

All procedures followed were in accordance with the Helsinki Declaration of 1975 as revised in 1983 following independent review and approval from the University of Florida Institutional Review Board. Data were collected from three groups totaling 80 adults (39 male) with every attempt made to age match controls and cohorts in this convenience sample. Twenty-five participants were HYA aged 18–29 years (mean age, 23 ± 6.8 years). Twenty-seven were HOA aged 56–81 (mean age, 70 ± 3.5 years) and 28 were status post-TL aged 48–80 (mean age, 60 ± 8.7 years) and time since surgery ranged from 1 to 30 years; (mean, 5 ± 4.5 years) (Table 1). Participants were screened via thorough review of their medical records prior to invitation to participate to exclude those with physician-diagnosed neurological or respiratory pathology, and during recruitment, participants were asked whether they had medical history of neurological or pulmonary disease. The cohort laryngectomy group included those without an active cancer diagnosis, no history of radiation exposure, and at least 1-year status post-TL.

**TABLE 1:** Demographic characteristics of each group (mean ± standard deviations).

	**Patients**	**Healthy controls**	
	**Laryngectomy**	**Older**	**Younger**
*n*	28	27	25
Male(s)	19	14	11
Age in years (mean ± SD)	46–80 (60 ± 8.7)	56–81 (70 ± 6.8)	18–29 (23 ± 3.5)
Years status post	1–30 (mean 5 ± 4.5)		

### Cough Procedures

Volitional and induced cough airflows were collected for all participants. For the HC groups, a facemask was placed securely over the nose and mouth, and connected to a spirometer (ADInstruments, MLT 1000) that digitized and recorded airflows via PowerLab (ADInstruments 8/30, model ML870, Australia, Pty Ltd.) to a computer for analysis with LabChart 7 (ADInstruments) at 1 k/s, low-pass filtered at 50 Hz. For the TL group, a neonatal facemask was securely fit over the stoma and connected to the spirometer, with airflow recorded to PowerLab. Instructions to elicit voluntary cough were to “cough as hard as you can, as if something went down the wrong way.” Three trials were completed with periods of tidal breathing collected between coughs. Induced cough was elicited with nebulized capsaicin (obtained by Formosa Pharmaceuticals) at three concentrations (50, 100, and 200 μM) as well as a saline control (0 μM capsaicin) over three randomized blocks. Capsaicin or control solutions were delivered via DeVilbiss nebulizer (DeVilbiss Model 646, Healthcare LLC, Somerset, PA, United States) positioned in-line with the facemask and spirometer. To aerosolize the solutions, a KoKo Dosimeter (nSpire Health Inc., Longmont CO, United States) was connected to the nebulizer and delivered pressurized air for 2-s durations upon detection of deep inspiration, with at least a 1-min latency between trials. Mass median particle diameters produced by the DeVilbiss model 646 average 5 μM. Following each capsaicin or control solution delivery, participants were asked to rate their urge to cough on a 0–10 ordinal scale using the modified Borg scale (Table 2), which is frequently utilized in the analysis of cough perception (Yamanda et al., 2008; Kanezaki et al., 2010; Hegland et al., 2011; Silverman et al., 2016).

**TABLE 2:** Borg Scale of urge-to-cough.

**Rating**	**Description**
0	None at all
1	Very slight
2	Slight
3	Moderate
4	Somewhat severe
5	Severe
6	
7	Very, very severe
8	
9	
10	Very, very, very severe (almost maximal)

Cough airflows collected for analysis included peak expiratory flow rate (PEFR), PEFRT, CVA, and post-peak phase integral (PPPI; Figure 1). The CVA variable is a metric of cough efficiency and is calculated as peak flow over rise time (CVA = PEFR/PEFRT). The PPPI metric captures sustained expiratory airflow after the peak has been reached and is functionally useful in preventing immediate re-inhalation of whatever particulates or materials have just been forcefully ejected from the airways.

### Cough Stimulus

Pharmaceutical grade powdered capsaicin was obtained from Formosa Pharmaceuticals, Inc. This pure chemical reagent was dissolved in 20 ml of ethyl alcohol (190 proof, 95% EtOH) to make a stock solution of 5 mM, which was subsequently diluted using a saline solution to make challenge concentrations 50, 100, and 200 μM. The final percentage of ethyl alcohol in the challenge solutions ranged from 0.95 to 3.8%.

### Statistical Analysis

Descriptive statistics including median, standard deviation, and confidence intervals were used to analyze the data, and given that these groups violated Levene’s statistic (*p* = 0.000) and were significantly heterogeneous per Breusch–Pagan heteroskedasticity (*p* = 0.000), non-parametric Kruskal–Wallis *H* test was used to detect significance differences between the independent variables (group and concentration) and the dependent sensorimotor cough variables (urge to cough, PEFR, PEFRT, CVA, and PPPI). Bonferroni and Tukey’s honestly significant difference test with *a priori* alpha set at 95% were used for *post hoc* analysis of group differences at each concentration and for each airflow metric.

## Results

**FIGURE 2:**
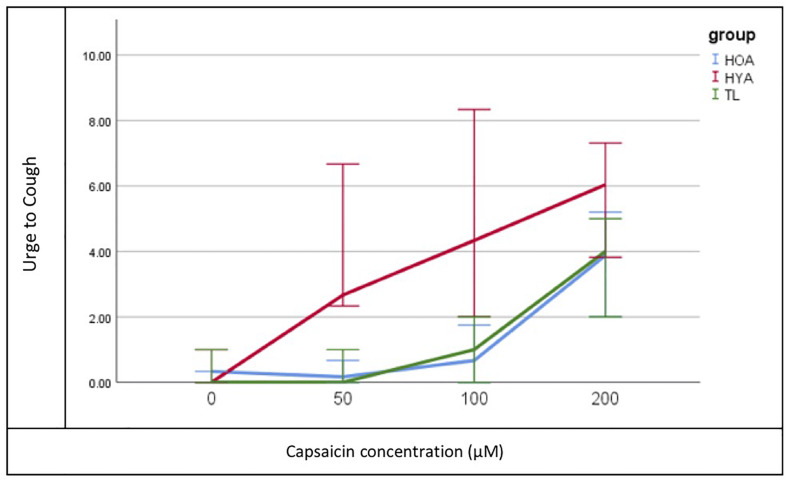
Median Urge to Cough (*y*-axis) plot for each concentration (*x*-axis) of capsaicin and each group (Total Laryngectomy (TL) green line; healthy older adult (HOA) blue line and healthy younger adults (HYA) red line). Error bars represent 95% confidence intervals.

Urge to cough following capsaicin-induced cough differed significantly between the three groups (TL, HOA, and HYA) χ^2^(2, *N* = 900) = 313.078, *p* = 0.001 (Figure 2). Descriptive statistics showed that median urge to cough increased in response to increase in concentration (saline or 0, 50, 100, and 200 μM were tested) and at 100 μM HYA reported median (±SD) urge as “somewhat severe” (4 ± 2) while both HOA and the TL group reported “very slight” urge to cough (1 ± 3 and 1 ± 2, respectively) (Table 3).

**TABLE 3:** Median urge to cough and total coughs produced by concentration for each group ± standard deviation.

**Concentration capsaicin (μM)**	**Healthy controls**		**Patients**
	**Younger**	**Older**	**Laryngectomy**
**Median urge to cough ± SD**			
0	0 ± 0	0 ± 1	0 ± 1
50	3 ± 2	0 ± 1	0 ± 1
100	4 ± 2	1 ± 3	1 ± 2*
200	7 ± 2	4 ± 3	4 ± 3
**Median total coughs produced ± SD**			
0	0 ± 0	0 ± 1	0 ± 0
50	3 ± 1	0 ± 1	0 ± 0
100	4 ± 2	0 ± 2	0 ± 1
200	4 ± 1	3 ± 2	2 ± 1

*Asterisk and box denote concentration at which groups were significantly different.*

Pairwise differences showed that HYAs had heightened perceived urge to cough at all levels while TLs and HOAs had comparable and reduced urge to cough (UTC). Bonferroni-adjusted comparisons of the three levels of concentrations (50, 100, and 200 μM) showed significant differences at 100 μM between HYA and TL [mean difference = 1.995, *t*(232) = 4, *p* = 0.000] and HOA [mean difference = 1.307, *t*(232) = 2, *p* = 0.046].

**FIGURE 3:**
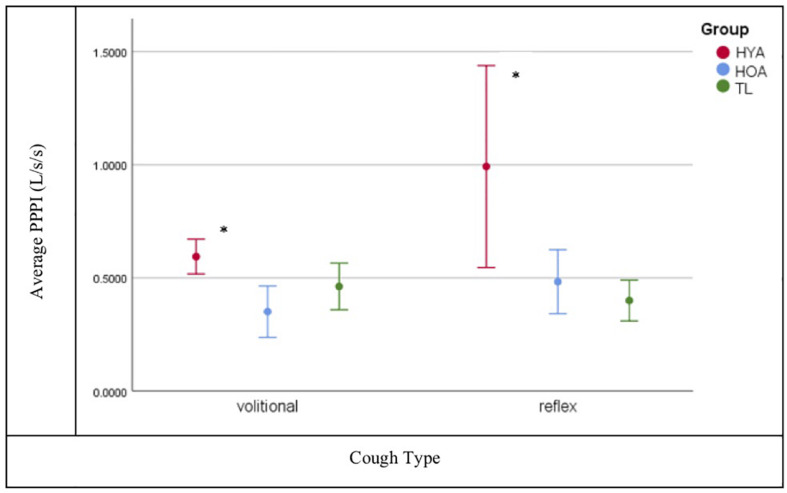
Post peak phase integral (PPPI) plot for volitional versus reflexive cough condition by group (Total Laryngectomy (TL) green line; healthy older adult (HOA) blue line and healthy younger adults (HYA) red line). *Denotes significant differences. Error bars represent 95% confidence intervals.

Tukey’s HSD *post hoc* analysis shows that all cough airflow measures (PEFR, PEFRT, and CVA) were significantly reduced for TLs (*p* = 0.001) compared to healthy controls with the exception of PPPI for which HOA and TLs were comparable (*p* = 0.998) under both volitional and capsaicin-induced cough conditions (Figure 3).

## Discussion

The hypothesis that post-TL subjects would have both reduced airflows and UTC was corroborated by these findings with one exception: PPPI was significantly reduced for both TL and HOA compared to HYA during both volitional and capsaicin-induced coughing. The PPPI metric represents sustained expiratory airflow after peak flow has been achieved and functionally prevents immediate re-inhalation of ejected particulates. The PPPI flow is sustained largely through activation of expiratory musculature and is in essence a prolonged “huff” after the cough. Under conditions wherein a weak cough is produced, as was the case with post-TL and HOA, prolonging the PPPI phase may represent a compensatory response for a reduced peak flow. Among the TL cohort, prolonged PPPI is likely the result of an absent glottal compression phase of cough, and for older adults, presbylarynx is a plausible etiology for their reduced cough airflows (PEFR, PEFRT, and CVA).

Urge to cough following capsaicin-induced cough was similar but reduced in both the post-TL and HOA groups relative to HYA. As mentioned, Fontana et al. (2002) found that cough thresholds were not significantly different between those with Parkinson’s disease and post-TL subjects in response to nebulized fog. Given that UTC typically precedes the motor cough response, we propose that UTC in the cohort of Fontana et al. (1999) was likely similar as well. Our results extend those of Fontana et al. (1999) in that UTC in both post-TL and HOA groups was blunted relative to HYA. Interestingly, differences in UTC sensation did not emerge at doses below 100 μM between post-TL/HOA and HYA, suggesting that doses of capsaicin below 100 μM may provide insufficient stimulation to elicit reliable urge to cough in both healthy controls and disordered populations.

A limitation of this study was the differing methods of tussigenic stimulant delivery between patients and controls given the anatomical differences status post-TL. The method of aerosol delivery in post-TL subjects relative to HOA and HYA was necessarily different, as post-TL subjects have complete separation of the airway and alimentary tracts and airway access can only be achieved through a stomal aperture in the neck. Lip-to-carina distance is 216–243 ± 11 mm (Stoneham, 1993; Varshney et al., 2011) in men and women, respectively, whereas distance from stoma to carina, likely within 60–90 mm, is considerably shorter, depending on extent of resection. Thus, the distance traversed by aerosolized stimulant is much shorter in the laryngectomy population and with fewer opportunities for deposition of inhaled particulates along the way, likely resulting in greater delivery of larger droplets (and volume) to the lower airways/carina than in our healthy control groups. Jet nebulizers similar to the one used for this study are known to deposit large droplet particulates to the upper airway. Thus, it might be argued that dose to structure (e.g., the lower airways) is not comparable between these groups and it may appear that while concentration remains constant, volume or micromolar mass median particle size (μM MM) delivered to the lower airways is not comparable. However, volume lost to the upper airways may be negligible (O’Callaghan and Barry, 1997) as most particulates greater than 10 μM MM are deposited in the upper airways. However, the dosimeter used in this study delivers particulates half that size, averaging 5 μM MM at a rate of 0.0025–0.005 ml/s (DeVilbiss Healthcare LLC, Somerset PA), of which greater than 65% is delivered to the large lower airways (O’Callaghan and Barry, 1997). It may therefore be assumed that at least 65% of the volume delivered to healthy controls (Table 4) reaches the lower airways, meaning the dose to structure between these two groups may not be comparable.

**TABLE 4:** Speculative dose to structure (lower airway) accounting for 35% deposition of particulates to the mouth and upper airways of healthy controls.

**Delivery method**	**Patients**	**Healthy Controls**

	**Stoma**	**Mouth**
Volume (ml per 2-s delivery duration)	0.005–0.01 ml	0.003–0.0065 ml

Reflex cough serves as an airway protective mechanism with urge to cough acting as a modulatory respiratory-related sensation indicative of cognitive processing with feedback loops to inform the cough network regarding magnitude of both stimulus and motor act (Davenport, 2009; Troche et al., 2014). Subjects post-TL lack both laryngeal sensory afferents and the need for deglutitory protection of the lower airways. Our data demonstrate that this population experiences reduced urge to cough in response to chemical stimulation with capsaicin aerosols. To date, the urge to cough has been extensively described as the “brain component” of the “cough motivation-to-action system” (Davenport et al., 2008) with input from sensory relay nuclei and the airway/pulmonary pump. This work elucidates the importance of laryngeal afferents in determining risk of airway invasion and informing cortical structures of the magnitude of that risk while highlighting evidence of redundancy throughout the system for eliciting airway protective behaviors. Further work with those lacking lower airway afferents, such as those status post double lung transplant, would serve as a unique comparison to our cohort and would establish the relative role in perceived urge to cough of upper versus lower airway afferents.

## Data Availability Statement

The datasets generated for this study are available on request to the corresponding author.

## Ethics Statement

The studies involving human participants were reviewed and approved by Institutional Review Board, University of Florida. The patients/participants provided their written informed consent to participate in this study.

## Author Contributions

AF and KW-H conceived the presented idea. AF and YM carried out the experiment. AF, KW-H, and DB contributed to the interpretation of the results. AF took the lead in preparing the manuscript. All authors provided critical feedback and helped shape the research, analysis, and manuscript.

## Conflict of Interest

The authors declare that the research was conducted in the absence of any commercial or financial relationships that could be construed as a potential conflict of interest.
